# A Christian Case for Farmed Animal Welfare

**DOI:** 10.3390/ani9121116

**Published:** 2019-12-11

**Authors:** Margaret B. Adam, David L. Clough, David Grumett

**Affiliations:** 1 Department of Theology and Religious Studies, University of Chester, Chester CH1 4BJ, UK; d.clough@chester.ac.uk; 2 School of Divinity, University of Edinburgh, New College, Mound Place, Edinburgh EH1 2LX, UK; David.Grumett@ed.ac.uk

**Keywords:** Christian ethics, animal ethics, farmed animals, Adam, Genesis, Noah, RSPCA, Singer, Peter, Regan, Tom, White, Lynn Jr.

## Abstract

**Simple Summary:**

It is now common to blame Christianity for broader society’s general inattention to the needs and comfort of animals in general, and farmed animals in particular. Critics claim that certain biblical themes and biblical passages form the foundation for an anti-animal position that has influenced Christians and wider Western society. This article concedes that Christianity has often been used to justify exploitation of animals, but argues that it is a mistake to consider Christianity inevitably opposed to concern for animals. It shows that Christians have been advocates for animals, notably in relation to the first legislation against animal cruelty in the early nineteenth century and the formation of the RSPCA. Finally, it proposes a framework for a Christian ethics of farmed animal welfare that could provide the basis for Christian action to reduce consumption of animals and shift to higher welfare sources of animal products.

**Abstract:**

It is now common to blame Christianity for broader society’s general inattention to the needs and comfort of animals in general, and farmed animals in particular. This critique of Christianity claims that certain biblical themes and particular biblical passages form the foundation for an anti-animal position that Christianity has imposed on Christians and on wider Western society. This article concedes that Christianity has often been used to justify exploitation of animals, but argues that it is a mistake to consider Christianity inevitably opposed to concern for animals. After reviewing the views of critics such as Lynn White Jr., Peter Singer, and Tom Regan, the article demonstrates the complexity of interpreting biblical passages and the possibility of readings that affirm the importance of treating animals well. It shows that Christians have indeed been advocates for animals, notably in relation to the first legislation against animal cruelty in the early nineteenth century and the formation of the RSPCA. Finally, it proposes a constructive framework for a Christian ethics of farmed animal welfare that could provide the basis for Christian action to reduce consumption of animals and shift to higher welfare sources of animal products.

## 1. Introduction

It is now common to blame Christianity for broader society’s general inattention to the needs and comfort of animals in general, and farmed animals in particular. Christianity, according to this presumption, teaches that humans should use animals for human satisfaction, that humans are more like God than animals, and that the needs and desires of humans supersede those of animals. This critique of Christianity claims that certain biblical themes and particular biblical passages form the foundation for an anti-animal position that Christianity has imposed on Christians and on wider Western society.

The authors of this article readily accept Christianity’s participation in the ill-treatment of farmed animals through the ages. Christians and others have granted authority to biblical interpretations in order to support their use and abuse of animals. However, we reject the simplistic account of Christianity as necessarily anti-animal; and we refute the presumption that Christians are bound to the exploitative domination of animals by one interpretation of a static text, across time and place, peoples and circumstances. This is patently false, as abundant evidence of Christian biblical interpretation, historical performances of interpretation, and current Christian support for farmed animal flourishing demonstrates. A more accurate account of Christianity and animal welfare notes the complex processes by which Christians engage with the Bible, each other, animals, and the world, by way of a multiplicity of interpretations, across a multiplicity of circumstances. Interpretations of Christianity are always accountable to the particular methods of interpretation, teaching, and ethical actions that their communities and traditions claim as authoritative. Interpretations always reflect contemporary scientific knowledge, socio-political-economic locations, cultural imaginations, and farming and eating practices. Christian biblical and doctrinal interpretation is always marked by continuity and difference.

The charge that Christianity is bad for animals neglects these resources and examples of Christian dedication to improving animal welfare. This essay shares some resources and some examples of what Christian support for animals looks like. Section 2 considers the current popular orthodoxy that Christianity is responsible for the poor treatment of farmed animals. Section 3 presents scriptural interpretations that demonstrate powerful Christian commitments to animal welfare. Section 4 describes historical lived interpretations of faith and a nineteenth century example of Christian animal advocacy. Section 5 sets out a contemporary assessment of farmed animal welfare in terms of flourishing, a Christian account of the best life possible for animals. Christian ethical engagement with farmed animal welfare illustrates the long-term Christian practices of interpreting scripture and engaging with doctrine for the benefit of animals.

## 2. Critics of Christian Understandings of Animals

The claim that Christianity is bad news for farmed animals has gained enough credibility amongst today’s animal advocates that it functions as a kind of orthodoxy: Christians only care about humans, and they think they have divine permission to exploit animals. Adherents to this critique note both the lack of Christian doctrine explicitly supporting farmed animal welfare and the negative influence that the writings of some Christian theologians have had on care for animals.

Lynn White, Jr., a 20th century medieval historian, offers a classic example of the charge that Western Christianity is responsible for the domination and exploitation of the natural world, including the animal world. White describes Christianity as ‘the most anthropocentric religion the world has seen’ [1] (p. 1205), and he supports this claim with his interpretation of Genesis 2, in which ‘a loving and all-powerful God’ creates all things, ending with Adam, who ‘named all the animals, thus establishing his dominance over them’ [1] (p. 1205). White, making uncritical use of ‘man’, understands this to mean that God ‘planned all of this explicitly for man’s benefit and rule: no item in the physical creation had any purpose save to serve man’s purposes’ [1] (p. 1205). According to White, Christianity justifies valuing nature solely in terms of its usefulness to ‘man’ by identifying ‘man’ as ‘not simply part of nature: he is made in God’s image’ [1] (p. 1205). White explains that, in Christianity, ‘man’s’ overriding purpose is to dominate nature, due to the foreshadowing in Adam of the image of Christ, and ‘man’s’ sharing in God’s transcendence.

The utilitarian philosopher Peter Singer has consistently made a similar, although more critical, case to White’s. The real significance of God making humans in his image, Singer suggests, is that humans make God in their own image [2] (pp. 186–208). Prominent in his argumentative arsenal is the critique of speciesism, which is the notion that the human species is intrinsically superior to all other species [3]. Singer thinks that Genesis presents humans as godlike, and as exercising a dominion of benevolent yet despotic rule over other species. He states that killing animals was permitted after the Fall, with God clothing Adam and Eve in animal skins on their expulsion from Eden, their son Abel killing sheep to offer to God, and God’s covenant with Noah after the flood, in which humans are given formal permission to consume animals as meat (Genesis 3:21, 4:1, 9:3). While acknowledging the scattered references in later Old Testament books to the harmony of humans and animals, Singer protests that there has been ‘no serious challenge to the overall view, laid down in Genesis, that the human species is the pinnacle of creation’ [2]. Further, Singer notes, the authority of Genesis as divinely revealed scripture justifies (to Christians) the irrational mistreatment of animals. He observes that the farm is a key location where such mistreatment may occur, detailing at length his concerns with United States chicken, egg, pig, dairy, and beef production systems [4] (pp. 21–67).

Like White and Singer, the animal rights philosopher Tom Regan connects the view that animals do not enjoy moral equality with humans and are not members of the moral community to the Genesis accounts of humans being made in God’s image and exercising dominion over all animals, and the story of all animals being given to Noah as food after the flood (Gen 1:24–28, 9:1–4) [5] (pp. 7–8, 127–128). Regan also describes how the medieval theologian Thomas Aquinas brings the Bible and Aristotle together. Regan recognizes, more than White or Singer, the diversity of the teaching on animals that Christian scripture contains, although he states that he is insufficiently expert to adjudicate within this area [6]. He does present alternative readings of key passages and themes, suggesting that human dominion, far from justifying animal exploitation, mandates humans to treat animals responsibly. He argues that Christ, following his resurrection and ascension, is related to the whole created order and not just to humans. However, Regan concurs with White and Singer in maintaining that, in practice, Christianity has promoted and sustained the human exploitation of animals, and that the dominant way in which Christians have treated animals has been exploitative [7].

White, Singer, and Regan have thus promoted the view that Christianity has created an intellectual and cultural climate that accepts and encourages the exploitation of animals. They argue that changing that climate requires resisting Christianity. We argue that Christianity provides a wealth of resources for supporting animal welfare. Section 3 and Section 4 offer examples of Christian textual and performative interpretations of scripture. Section 5 proposes a Christian ethical framework for improving farmed animal welfare.

## 3. Interpreting the Bible as Christian Animal Advocates

White, Singer, and Regan are correct that that Christians—and others—have used Christian scripture and theology to justify animal exploitation, but it is incorrect to judge that this is the necessary or only legitimate interpretation of Christian belief. The representation of Christianity as anti-animal neglects Christianity’s resources of biblical interpretation, teaching, and action that greatly value all creatures.

The charge that Christianity is anti-animal relies on one interpretation of a few select biblical passages, while neglecting others. That narrow reading limits biblical interpretation to a clumsy literalism. It simply is not the case that all Christians, all people, make sense of the Bible in the same way. Ideology, imagination, information, experience, era, and cultural systems all influence communities’ interpretations; and a host of distinctly Christian interpretations, arguments, and actions support and promote the health and well-being of animals.

The book of Genesis begins with two accounts of creation. These are not histories as history is now conceived; there is no claim of verifiable accuracy or documentary evidence. They are stories for receiving and making meaning, in faithful communities. In each account, God creates and orders the cosmos, the earth, all creatures—human and animal. In each account, humans and animals share creatureliness and habitat. In the first, God gives all green things as food for all creatures (no human or animal is to eat any other human or animal). In the second, God gives humans a garden for food (and it is not clear what animals should eat). In each account, God establishes a relationship between humans and other animals that, in some way, reflects God’s care for creatures. In Genesis Chapter 1, God grants humans dominion over all living things in the sea, in the air, and on the earth. In Genesis Chapter 2, God instructs Adam to name each of the animals. Christians make sense of dominion, the ramifications of naming, and the creation diet in multiple ways [8]. In the biblical narratives of ancient monarchies, a good and faithful king improved the welfare of the people; good human dominion might improve the welfare of animals. One interpretation that takes dominion as human leadership into shared creaturely peaceful existence supports vegetarian diets as ideal [9]. Dominion that reflects Jesus’ servant–king status rather than domination supports humble duty to animals’ wellbeing [8] (pp. 222–224). Still other understandings of dominion highlight humanity’s responsibility to reflect God’s compassion [10] and the exercise of God’s love through neighbourly love for animals [11].

In the second creation story, God presents the animals and birds to Adam as potential helpers and partners, and directs Adam to name them. Adam does, but the story continues by noting that none of these animals makes a suitable helper or partner (Gen 2:18–20), and God creates Eve from Adam’s rib. Many scholars have focused on the inadequacy of the animals and birds as helpers and partners, applying to this second story the human dominion over animals of the first creation account (1:26), which is from a different source [12,13]. Some champions of human superiority read Adam’s naming of the animals as the origin of language, in which human action designates and classifies animals and the wider physical world [14,15]. Alternatively, the naming can be compared to the way parents name their children: Adam identifies the animals as, in some sense, family members [16] (p. 1006). Indeed, Adam’s naming of the animals can be interpreted as a lens for reading the whole of Genesis 1–3 in relational terms: Chapter 1, humans are created as part of the same process in which animals are created; Chapter 2, a human is formed as a living being out of the dust of the ground (2:7) and then names the animals; Chapter 3, both humans and animals are held responsible for disobeying God’s intrusions and eating the fruit from the tree of the knowledge of good and evil. Accordingly, a relationship between humans and animals founded on shared creatureliness and mutual recognition better characterizes the image of God in humanity (1:27) than the estrangement of humans from animals [16] (p. 1006).

Along these lines, the creation stories’ vegan dietary directives cohere with the shared, non-violent, creatureliness of humans and animals [17]. The imagination of a non-violent coexistence contrasts sharply with the apparently natural existence of conflict and the evolutionary survival-of-the-fittest species-development. This should not be surprising: biblical stories of creation differ in genre from philosophical discourse and from evolutionary science; but difference in genre does not require competition for truth. In this case, Christian animal advocates can draw on the creation stories’ plant-based diets as an expression of God’s will for non-violent relationships between humans and other creatures. Christian ethics is attentive and responsive to theological assessments of God’s will, however implausible it may seem for humans to achieve that will.

The story of Noah (Gen 6:9–9:17) provides more material for considering human relationships with animals. Singer and Regan present the story negatively, because it authorizes meat-eating (Gen 9:1–3), but the story is much more complicated than that. In the previous two chapters of Genesis, Noah preserves all the terrestrial animals and all the birds on the ark during the flood. After the flood, he kills some of the animals to offer to God and for consumption as meat, despite having protected the animals within the ark for just over a year. Noah, according to Norman Wirzba’s theological interpretation [18] (pp. 115–122), offers to God a costly sacrifice that is characterized not simply as a violent act of killing but as an integral part of wise, attentive, skilled animal management, in which a duty of care is diligently exercised. Noah’s care for the animals mirrors God’s care, and his offering of some animals to God affirms that all animals come from God and belong to God. We might imagine the loss felt by Noah when offering up his animals to God as comparable to the loss felt by many keepers of animals when sending them for slaughter. Other interpretations note that God, having seen humanity’s inability to refrain from violence, grants humans permission to eat some meat, provided they follow strict guidelines that honour the life that is killed. Thus, the permission is less a divine retraction of creation veganism and more an allowance for persistent human sinfulness [19] (pp. 91–99) [20].

Later in the Old Testament, there are direct requirements in relation to animal welfare. Sabbath regulations protect animals used for draught labour alongside human beings (Exodus 20:8–11, 23:12; Deuteronomy 5:14) and even affirm the importance of providing for wild animals (Leviticus 25:6–7). First-born male livestock must remain with their mothers for seven days before being sacrificed (Exod 22:30), and mothers and cows or ewes should not be slaughtered with their young on the same day (Lev. 22:28). Donkeys must be released from being trapped under their burdens (Exod 23:4–5; Deut 22: 1–4), kids may not be boiled in their mothers’ milk (Exod 23:19; Deut 14:21), a mother bird should not be taken with her fledglings or eggs (Deut 22:6–7), and oxen should not be muzzled when treading the grain (Deut 25:4). These texts have been interpreted by Jews and Christians as requiring concern for animal welfare.

Christians who promote animal welfare often look to the prophecies of Isaiah that describe a peaceable kingdom in which God’s ordering of creation is fulfilled (Isaiah 11:1–9; 65:17–25). Isaiah announces that the end, the goal, of creation, will be characterized by the harmonious co-existence of all creatures. Animals we know as carnivores will live and share straw with herbivores; a human child will play safely by an asp; harmony—not interspecies violence—will abide. Christians who challenge human reliance on killing animals for survival use these passages to stretch imaginations. If it is possible to imagine a prophetic vision of God’s reconciled creation, perhaps it is possible to reconsider conventional approaches to farming animals, or modify one’s diet, as small gestures in the direction of that vision. Paul, writing to the community of early Christians in Rome, recognizes the difficulty of living between peaceable kingdom hope and the decidedly non-peaceful world we know (Romans 8: 18–25). He describes that hope as the labour pains of the entire cosmos, longing together for creation’s ultimate reconciliation. Christian ethics of farmed animal welfare addresses how to live now, in between, while hoping for God’s kingdom to come [21].

Jesus addresses the priorities of human daily life, here and now, with a reminder of God’s constant care of all creatures (Matthew 6:26–34): worrying about the future, about having enough food and clothing, distracts people from faithfulness. Jesus points to the birds who do not grow, harvest, or store crops, and they still do not worry, because God provides for them in every way. ‘Are you not of more value than they? And can any of you by worrying add a single hour to your span of life?’ (Matt 6:26–27, New Revised Standard Version). Those determined to underscore human superiority over animals may read this passage as affirming the higher and lower positions of humans and animals. Those who understand Jesus to be talking to human creatures less faithful and more self-absorbed than birds may read the passage as chastising humans and commending the example of the birds who never fail to give glory to God by their very existence [22] (pp. 43–44).

These are but a few of the ways Christians understand scripture in terms of animal welfare. Interpretive imaginations will continue to shift as more people learn about animal sentience and cognition, about what happens on farms and in slaughterhouses, and about how the market for inexpensive meat affects farmers and farmed animals. Christian communities hold the responsibility of interpreting scripture in the light of both earlier interpretations and present circumstances.

## 4. Examples of Christian Advocacy for Animals

Beyond the realm of variant textual interpretations, Christianity’s heritage of animal advocacy offers a steady stream of faithful people whose lives exhibit their scriptural interpretation about relationships with animals. The church tells and retells stories about biblical characters and pre-modern saints, often with extravagant embellishment, the better to illustrate the exceptional holiness of the figures. While there may be scant historical evidence about some of these saints themselves, vast numbers of people across centuries celebrate them and the faithfulness their stories illustrate. Many of the saints had special relationships with animals and were able to communicate with them, share home and food with them, heal them, protect and rescue them. Others were protected and rescued by friendly animals. Many did not eat meat; some only ate herbs and honey. Together, these stories and their popularity present a body of evidence that, across centuries, Christianity has recognized as exceedingly holy Christians who lived closely with animals in ways that reflect the creation stories and anticipate images of the peaceable kingdom.

Two examples from the Old Testament show people receiving God’s care through the agency of helpful animals. Ravens feed Elijah food when he passes through an area suffering a drought that he had prophesied (I Kings 17:2–6). Balham’s donkey speaks necessary (and intelligible) words of chastisement and redirection so that his owner might notice the angel of the Lord in front of him (Numbers 22). In early Christianity, St Chrysostom advocated abstinence from meat and preached, ‘The Saints are exceedingly loving and gentle to mankind, and even to brute beasts…surely we ought to show them [animals] great kindness and gentleness for many reasons, but, above all, because they are of the same origin as ourselves’ [23]. St Anthony rescued from satanic possession a pig, who then accompanied Anthony everywhere (as recounted in an eighteenth century ballad) [24]. St Modestos healed a poor woman’s ailing oxen [25]. St Cuthbert protected birds from hunters and otters warmed his feet [26]. St Melangell persuaded a hunter prince to establish a sanctuary for people and animals, after the hunts’ hares found refuge with her, and the dogs retreated, awed by her presence. [27]. St Francis [28], perhaps the best known saint to have friendships with animals, followed in the footsteps of a host of predecessors, and was followed by many more.

Abstinence from meat hasbeen a normative practice of devotion throughout the church from its beginning: weekly and in penitential seasons for lay people, more frequently and more severely for monks and nuns in religious orders, and sometimes to the extreme for saintly hermits. Versions of these practices continue today. Where an explicit reason is given for abstinence from food, usually it is an ascetic concern to turn away from distracting pleasures and turn toward God. It can also reflect a desire to live in accordance with the peaceable creaturely existence envisioned in Genesis and Isaiah (discussed in Section 3). These examples might serve as a reminder that Christians have long embodied their faith commitments in what, when, and how much they eat. The early Reformers moved away from some traditional practices of piety as they critically assessed and established alternatives to Catholic marks of piety. A number of animal-appreciative Nonconformists declared the goodness of animals, contradicting both the established church and fellow Nonconformists. John Calvin and the Westminster Confession presented animals as members of creation through which God’s glory is revealed. The Puritans, John Trapp, Thomas Watson, and Stephen Charnock, all celebrated animals for fulfilling their creaturely purpose, to glorify God [29].

The eighteenth century’s rise in vegetarianism was fueled by the appeal of Romanticism and Nature to the wealthy, by the poor’s inability to afford much meat, and by new theories about vegetarianism’s potential health benefits. In the following century, a Christian movement in the UK made headlines and a lasting contribution to animal advocacy. The Christian origins of the Royal Society for the Prevention of Cruelty to Animals (RSPCA) are not well known now, among animal advocacy critics of Christianity or among most Christian communities. This example of socio-political activism, grounded in biblical interpretation and Christian teaching, serves both as evidence of effective Christian animal advocacy and as inspiration for contemporary Christian support for animal welfare.

In the United Kingdom during the nineteenth century, Christians played a key role in putting animal welfare on the moral, social, legal, and political agendas by founding what became the RSPCA. They were motivated by earlier theological accounts of the Christian significance of animals and by the belief that Christian faith should inform the way society was ordered. The latter informed Christian campaigns on other social issues in the same period, such as for the abolition of the slave trade. First, the 1822 Cruel Treatment of Cattle Act empowered magistrates to fine anyone found to have beaten, abused, or mistreated cattle or sheep, and if they could not pay, or refused to pay, to imprison them [30] (pp. 285–288). The support of clergy and evangelical MPs helped pass this and other legislation [31] (pp. 27–29) while evangelicalism’s political power was reaching its height in the 1830s and 1840s [32] (pp. 203–205) [33] (pp. 204–209). In 1824, the Society for the Prevention of Cruelty to Animals (SPCA) came into being. Among the Society’s founding members were three Anglican clergy, including the Revd Arthur Broome, who became the Society’s first chair. Five of the ten founder members of an allied organization—the Association for Promoting Rational Humanity towards the Animal Creation (APRHAC)—were also clergy, with Anglicans predominating [34] (p. 29).

Broome resigned from his parish to focus on his SPCA work, which had become his primary Christian ministry, even spending time in a debtor’s prison as his position was unpaid and he had used his own money to fund the organization. Chien-hui Li writes of the constellation of British animal welfare organizations in this period that the ‘Christian tradition quite overwhelmingly prevailed over other possible sources of influence and became its principal source of identification, legitimation, and inspiration’ [34] (pp. 30–31). Sermons were a key means of influencing public opinion, along with Christian educational and publicity materials. Annual meeting statements, exhortations, and resolutions displayed clear Christian content. Prayers and hymns were part of meetings. The SPCA’s Christian principles contrasted with secular radicalism’s use of ancient Greek thought to support veganism and reforms to farming and slaughtering.

Farmed animal welfare was a key concern of the SPCA from its inception, as the 1822 Cruel Treatment of Cattle Act indicates. The Society campaigned or prosecuted on issues including calf bleeding for veal, livestock transport by rail and steamers (including unloading), inhumane slaughter methods, long travel distances to London markets, the overstocking of cattle markets to reduce prices, leaving unmilked cows in market to increase their price, the dehorning of cattle, and the nose-branding of sheep [31] (pp. 133–134, 146–147, 173–190). In 1840, after gaining the support of Queen Victoria, the SPCA became the RSPCA. In its early decades, its promotion of farmed animal welfare was motivated by a Christian ethics of mercy, kindness, and compassion [34] (pp. 31–36) that formed part of a wider project of civilizing the working class and raising its moral standards [35] (pp. 125–57) [36] (pp. 20–24). The detail of the Christian justifications given for its work, and their biblical grounding, may be seen in three published entries for the SPCA’s 1837 essay prize. A sum of £100 (about £11,000 in today’s money) was offered for the best essay on the religious basis for human obligation towards animals. Each submission includes a chapter on the biblical basis for protecting animal welfare, and the approaches are similar. There had been minority pro-welfare readings of the Bible in the 18th century and the competition may have revived these [37]. The competition entries demonstrate how the Bible can be read and presented to promote animal welfare in a particular context.

Essay themes include God’s watchful care for all creatures, and the correlation between human righteousness and merciful treatment of animals. Each author supports his arguments that God values and attends to each and every creature with interpretations of biblical passages: work animals need rest and nourishment (Exod 20:8–11, 23:12; Luke 13:15); even little sparrows and lambs receive God’s protection (Matt 10:29, Luke 12:6, 2 Samuel 12:1–6); God’s post-flood covenant includes all of creation (Gen 9:9–12). Further, they assert that merciful care of animals marks a person’s righteous character: know your animals’ needs (Proverbs 12:10); rescue animals in need (Deut 22:1–4); show mercy to all people and animals (Luke 6:36, Matt 5:7).

Most descriptions of the passing of legislation against cruelty towards animals in the early- to mid-nineteenth century exaggerate the contributions of Jeremy Bentham’s utilitarianism and ignore the roles of Christian arguments and evangelical Christians in Parliament [38] (pp. 4–9). The origins of the RSPCA demonstrate that utilitarianism was not the only intellectual or practical impetus for farmed animal welfare concern and that Christian ethics may even have been more important. During the second half of the nineteenth century, as acceptance of the RSPCA spread, its underpinning Christian ethos became gradually more secularized, but the framework of mercy, kindness, and compassion remain today.

The early RSPCA grounded responsibility for animals in the belief that humans are distinct from and superior to animals. Much of recent animal advocacy resists hierarchical accounts of humans and other animals, preferring an egalitarian understanding and a focus on what humans and animals have in common. The Christian belief that the primary distinction is between God (creator of all creation) and creatures (including all humans and animals) supports an emphasis on the shared creaturely status of all creatures, rather than on human superiority. At the same time, ethics addresses human action. Nobody is suggesting that farmed animals should fight for reform in farming systems; humans bear the responsibility to improve farmed animal welfare. A Christian account of creaturely relationships places the agency for animal care in human hands. The presumption of human superiority may lead to human use and abuse of animals; but the claim that all fellow creatures are equal risks relieving humans of their agency to assist those in need. Christian ethics of farmed animal welfare identifies human distinctiveness as the agency to reflect, with humility, God’s mercy, kindness, and compassion in caring for farmed animals.

## 5. A Proposed Framework for the Christian Ethics of Farmed Animal Welfare

In the previous section, we have argued that there are reasons to reconsider simplistic and negative assessments of the contribution Christianity has made to understandings of animal welfare. In this section, we turn to the constructive task of setting out a Christian framework for considering farmed animal welfare. Our focus is on farmed animal welfare because this is an obvious priority in relation to other human uses of animals on the basis of scale and impacts, though the framework we set out is applicable to contexts beyond animal farming [39]. Such a framework could be used to guide the policy and practice of churches and other organizations seeking to reflect their Christian commitments.

The starting point for the Christian ethics of farmed animal welfare we propose picks up strands from the tradition noted in the previous section to affirm that, for Christians, the lives of all creatures have value because they are created by God as ends in themselves and to glorify God in their flourishing. Christians recognize that all creatures exist in utter dependence on God and on one another. No creature exists merely as the means to the wellbeing of another. God delights in the flourishing of a universe of diverse creatures, and Christians are given the high calling of being images of this loving God in their relationships with fellow creatures. Christians, therefore, have strong reasons to seek to enable the flourishing of fellow creatures where possible. The particular modes of life, capacities for happiness and suffering, and other vulnerabilities of fellow animal creatures give Christians especial reasons for being concerned about their wellbeing. The theological basis for this understanding of animals is developed in Clough [40]. This Christian understanding of creaturely flourishing has common features with other teleological approaches that are attentive to the goal of creaturely life, such as Aristotelian ethics, but departs from them by identifying the goal of creatures in relationship to their Creator.

Concern for the flourishing of farmed animal creatures requires attention to the question of what constitutes a good life for particular species. Answering this question depends on detailed knowledge concerning the modes of life and preferred behaviours of farmed animals. It encourages appreciation of the whole of the life of animals, raising issues neglected in narrower understandings of animal welfare, such as whether farmed animals are able to experience maternal care, life in family groups, and growth to maturity. An assessment of whether farmed animals are living lives in which they are flourishing will include regard for negative experiences such as hunger or distress but also recognizes the importance of positive dimensions of flourishing, such as the ability to forage, graze, or exercise choice between areas with different characteristics. Promoting flourishing in this sense will mean opting for a mode of life providing positive dimensions of flourishing even at increased risk of some distressing experiences, such as in well-designed free-range environments.

It would be of interest to explore both common ground and differences between this proposed Christian foundation for the ethics of farmed animal welfare and other frameworks for animal ethics such as animal rights, utilitarianism, social contract, virtue ethics, feminist ethics of care, or those drawing on Aristotle. We are not pursuing that task here because our primary concern is to open a space for a constructive engagement between Christian ethics and farmed animal welfare. Our argument here is not that this Christian framework is preferable to other approaches to animal ethics, but that it may contribute additional perspectives to the field of approaches and that it is helpful in encouraging and enabling Christians to make connections between their faith commitments and practice in relation to farmed animals. Many of the most pressing actions that follow from the Christian framework we propose will encourage Christians to make common cause with animal advocates who have different starting points.

A useful next step after setting out the foundation of a concern for the flourishing of farmed animals is to recognize the major shifts in the ways animals have been used for food in the UK. Hundreds of years ago, there was a gradual shift from nomadic herding to enclosed farming. The Industrial Age of the eighteenth century altered animal farming with new equipment and breeding techniques. Since the Second World War, the pace and scope of technological innovation and intensification of animal agriculture has developed rapidly. Today, a confluence of socio-economic developments motivate and sustain the development of unprecedentedly large intensive farming systems. Most intensively farmed animals have been selectively bred for efficient production at the cost of their capacity to flourish more generally and are kept indoors in impoverished monotonous environments that do not allow many preferred species-specific behaviours. Chickens and most dairy calves do not meet their mothers, depriving both mother and offspring of a very significant component of a good life. Broiler chickens, most dairy calves, and most pigs and lambs live short lives and do not reach maturity. The high levels of production enabled by these systems of farming primarily benefit higher level corporate managers and retailers. This unprecedented increase in farmed animal productivity correlates with current consumer expectations of inexpensive meat products for daily meals. Many individuals and households eat predominantly meat-based prepared food, at home, from take-aways, and at restaurants, several times a week. Fewer and fewer consumers have seen farmed animals as they are raised, and the idyllic image of the small family farm with freely roaming animals persists, long after it ceased to represent the norm. Advertisements encourage irresistible desires for farmed animal products, often associating consumption of meat with masculinity [41].

The juxtaposition of a Christian rationale for being concerned about the flourishing of farmed animals with a recognition that modern industrial animal agriculture fails to allow such flourishing leads directly to the judgement that Christians have strong reasons for reconsidering their involvement with this practice as producers, retailers, and consumers. The ethical concerns raised by animal agriculture in the early twenty-first century are far greater than those that gave Christians cause for concern in the early nineteenth century. It is striking, therefore, that to date, Christians have not mustered a comparable response. This is in spite of some attempts to raise concern for animals as an issue for Christians, such as the work of Linzey and Clark [42,43]. Most Christians promoting farmed animal welfare engage in their advocacy with secular organisations outside the church. Farmed animals are rarely mentioned in church contexts, and meat still dominates church community meals without regard to the conditions of the animals before or during slaughter.

There are straightforward practical actions that follow from the acceptance of the analysis generated by the Christian framework for the ethics of farmed animal welfare we propose. First, steps should be taken to reduce overall consumption of farmed animals. This is necessary because it is not possible to raise animals in ways that give them more opportunities to flourish at anything like current production levels. A recent report calculates moving to pasture-fed beef cattle in the US would reduce production by 73% [44]. Bringing an end to the intensive rearing of pigs, broiler chickens, and dairy cows in indoor sheds is likely to require similar reductions. It is notable that reducing overall consumption of farmed animals would also bring benefits of reducing the contribution of animal agriculture to habitat loss causing wild animal extinctions, improving human food and water security, improving human dietary health, and bringing environmental benefits of reducing deforestation, greenhouse gas emissions, and pollution [39] (pp. 54–59). Christians can take action to reduce consumption of animal products at individual and corporate level, through shifts towards more plant-based foods domestically, in considering food served by churches, and in catering policies of organizations with Christian foundations.

The second straightforward practical action that follows from the Christian ethical analysis we present is to source remaining animal products from producers who allow farmed animals more opportunities to flourish. This can be an incremental approach, beginning with more simple changes, such as not using eggs from caged hens, attending to the various certification and grading schemes that assess farmed animal welfare, and then looking for opportunities to source animal products from suppliers using heritage or rare-breed animals that have not been subjected to modern selective breeding. Again, action can be taken to improve sourcing domestically, within church communities, and in organizational-level decisions about catering.

One reason some Christians are cautious about the reduced consumption and higher welfare sourcing of farmed animal products we propose is their acute awareness of the situation of farmers. Most livestock farmers are doing their best to care for their animals in the context of very challenging economic circumstances and uncertainty about the future of their business. For many, these external factors cause social isolation and high levels of stress. Understandably, farmers and those who support them can feel threatened by and resistant to claims that there need to be significant changes in the ways animals are raised for food. A Christian engagement with farmed animal welfare must attend to the wider context of the flourishing of farmers and farm workers alongside the flourishing of animals. At the same time, this does not weaken the case for a transition towards raising fewer animals and giving the remaining animals more opportunities to flourish. There is a broad and widening consensus of the need for this transition (see, for example, the recent RSA report [45]). The industry must undergo a transition, with the cooperation of retailers and consumers. During this transition, we must attend to the wellbeing of farmers alongside farmed animals. Churches should listen to and support farmers as they determine how to make a living from producing food in this changing context.

Another concern Christians raise is that producing fewer and better animal products will raise the prices of animal products, which will negatively affect food access for those on low incomes. There are three key reasons that this important concern does not weaken the case for rethinking animal agriculture. First, on a global level, current patterns of raising animals exacerbate human food insecurity and raise food prices by feeding food that humans could consume to farmed animals. Over one-third of global grain output is fed to livestock, rather than consumed directly by humans. This is a grossly wasteful practice, with a calorific efficiency of less than 10% [46]. Second, while it is true that subsidies for animal agriculture mean that in some urban contexts highly processed animal products are the cheapest food available, that food constitutes an unhealthy diet with high disease risks for populations that are disproportionately poor and non-white. A growing literature interrogating the intersection between poverty, racial inequality, and food justice makes clear that the products of industrial animal agriculture are part of the problem here, not part of the solution (see, for example, Harper [47]). Third, current practice in animal agriculture subjects workers on farms and in meat-processing plants—who are disproportionately female, migrant, non-white, and poor—to unsafe working conditions with negative impacts on their physical and mental health [39] (pp. 54–56). A transition towards fewer and better animal products must ensure access to these products for those on lower incomes, but the overall impacts of the current system are at least as problematic for the poor as for the more wealthy.

In this section, we have outlined a Christian framework for the ethics of farmed animal welfare that provides motivation and guidance for Christians to rethink their involvement with the current practice of industrialized animal agriculture and to recognize faith-based reasons for reducing overall consumption of animals and moving to higher welfare sources for remaining animal products. We have identified a concern for the flourishing of fellow animal creatures as the starting point for this framework. This focus might be applicable to human uses of animals for research, labour, textiles, sport and entertainment, and for companion animals [39]. We argue that attention to farmed animals should be the primary concern, on grounds of scale and impact.

## 6. Conclusions

After summarizing the positions of Christianity’s critics in Section 2, we developed the Christian case for farmed animal welfare in three stages. Section 3 demonstrated the complexities involved in the Christian interpretation of biblical texts, which give reasons to be very cautious about taking particular texts out of context to justify a particular view of how animals should be treated. It also demonstrated the potential for readings of biblical texts that celebrate God’s love for animals and human responsibility for caring for them. Section 4 showed that Christians have, in fact, interpreted biblical texts to affirm the importance of concern for animals, with a particular focus on the Christian arguments used for the first UK legislation against cruelty towards animals in the early nineteenth century, and the significance of Christianity in the formation of the organization that became the RSPCA. Section 5 set out a particular way of drawing on this scriptural and historical inheritance to make the case that Christians have strong faith-based reasons to be concerned about industrialized animal agriculture. Christians should act to reduce consumption of animal products and source animal products from higher welfare sources.

We have conceded with regret that critics such as Lynn White Jr., Peter Singer, and Tom Regan are right in claiming that Christianity has been used to support the human exploitation of animals without adequate regard for their wellbeing. We have argued that it is wrong to jump from that claim to the judgement that Christianity is inevitably an enemy of concern for animals. This latter judgement is problematic for two reasons. First, it is inaccurate, because it fails to recognize that Christianity has often been used to promote concern for animals. Second, it is unhelpful, because it suggests to both Christians and non-Christians that faith commitments give Christians no reason for being concerned about animal welfare. In this article, we have made the case that Christianity can be a strong ally in efforts to promote farmed animal welfare. We hope the argument will be persuasive both among Christians and among non-Christian animal advocates in order to make possible new coalitions working for advances in animal welfare generally, and farmed animal welfare in particular.
